# [Bis(pyridin-2-yl) selenide-κ^2^ *N*,*N*′]tetra­chloridotin(IV)

**DOI:** 10.1107/S160053681202586X

**Published:** 2012-06-30

**Authors:** Gunay Z. Mammadova, Zhanna V. Matsulevich, Vladimir K. Osmanov, Alexander V. Borisov, Victor N. Khrustalev

**Affiliations:** a Baku State University, Z. Khalilov St 23, Baku, AZ-1148, Azerbaijan; b R.E. Alekseev Nizhny Novgorod State Technical University, 24 Minin St, Nizhny Novgorod, 603950, Russian Federation; c X-Ray Structural Centre, A.N. Nesmeyanov Institute of Organoelement Compounds, Russian Academy of Sciences, 28 Vavilov St, B-334, Moscow 119991, Russian Federation

## Abstract

The title compound, [SnCl_4_(C_10_H_8_N_2_Se)], was obtained by the reaction of 2,2′-dipyridyl diselenide with tin tetra­chloride. The Sn^IV^ ion is coordinated by two N atoms [Sn—N = 2.266 (2) and 2.274 (2) Å] from the bis­(2-pyrid­yl)selenide ligand and four chloride anions [Sn—Cl = 2.3717 (6)–2.3939 (6) Å] in a distorted octa­hedral geometry. The central six-membered chelate ring has a boat conformation with the Se and Sn atoms deviating by 0.692 (3) and 0.855 (3) Å, respectively, from the mean plane through the remaining four ring atoms. The pyridine rings are inclined to each other by a dihedral angle of 49.62 (8)°. The crystal packing exhibits short inter­molecular Se⋯Cl contacts [3.5417 (7) and 3.5648 (7) Å], weak C—H⋯Cl hydrogen bonds and π–π stacking inter­actions between the pyridine rings with a centroid–centroid distance of 3.683 (3) Å.

## Related literature

For the crystal structure of the 2,2′-dipyridyl-selenide ligand, see: Dunne *et al.* (1995 ▶). For the crystal structures of related compounds, see: Tresoldi *et al.* (1992 ▶); Kondo *et al.* (1995 ▶); Blake *et al.* (2002 ▶); Teles *et al.* (2006 ▶); Zhao *et al.* (2007 ▶); Wriedt *et al.* (2008*a* ▶,*b* ▶,*c* ▶).
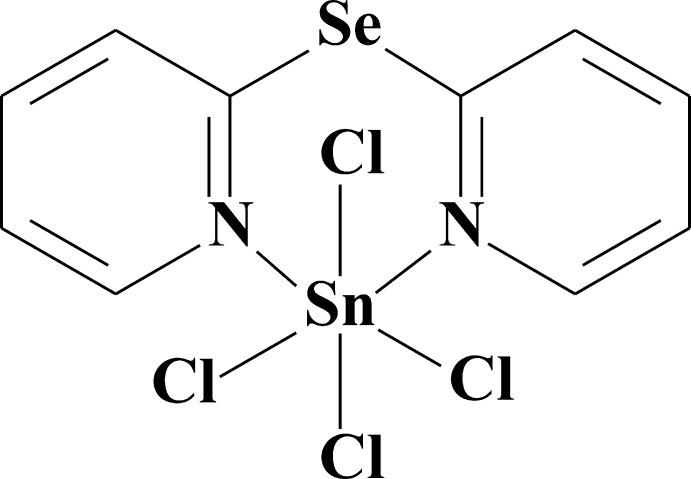


## Experimental

### 

#### Crystal data

[SnCl_4_(C_10_H_8_N_2_Se)]
*M* *_r_* = 495.63
Monoclinic, 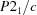

*a* = 8.0835 (4) Å
*b* = 12.2153 (5) Å
*c* = 14.4710 (6) Å
β = 101.208 (1)°
*V* = 1401.65 (11) Å^3^
*Z* = 4
Mo *K*α radiation
μ = 5.16 mm^−1^
*T* = 100 K
0.30 × 0.24 × 0.15 mm

#### Data collection

Bruker SMART 1K CCD diffractometer
Absorption correction: multi-scan [*SADABS*; Sheldrick, 1998 ▶) *T*_min_ = 0.306, *T*_max_ = 0.511
16245 measured reflections
4096 independent reflections
3723 reflections with *I* > 2σ(*I*)
*R*_int_ = 0.026

#### Refinement

*R*[*F* ^2^ > 2σ(*F* ^2^)] = 0.025
*wR*(*F* ^2^) = 0.058
*S* = 1.00
4096 reflections
163 parameters
H-atom parameters constrained
Δρ_max_ = 1.64 e Å^−3^
Δρ_min_ = −1.10 e Å^−3^

### 

Data collection: *SMART* (Bruker, 1998 ▶); cell refinement: *SAINT* (Bruker, 1998 ▶); data reduction: *SAINT*); program(s) used to solve structure: *SHELXTL* (Sheldrick, 2008 ▶); program(s) used to refine structure: *SHELXTL*; molecular graphics: *SHELXTL*; software used to prepare material for publication: *SHELXTL*.

## Supplementary Material

Crystal structure: contains datablock(s) global, I. DOI: 10.1107/S160053681202586X/cv5309sup1.cif

Structure factors: contains datablock(s) I. DOI: 10.1107/S160053681202586X/cv5309Isup2.hkl

Additional supplementary materials:  crystallographic information; 3D view; checkCIF report

## Figures and Tables

**Table 1:** Hydrogen-bond geometry (Å, °)

*D*—H⋯*A*	*D*—H	H⋯*A*	*D*⋯*A*	*D*—H⋯*A*
C3—H3⋯Cl3^i^	0.95	2.79	3.3965 (18)	122
C8—H8⋯Cl2^ii^	0.95	2.83	3.3126 (18)	113

Symmetry codes: (i) 
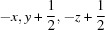
; (ii) 
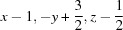
.

**Fig. 1.**
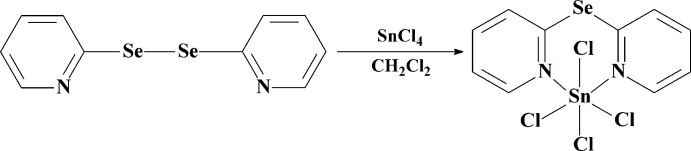
Reaction of 2,2'-dipyridyl diselenide with tin tetrachloride.

**Fig. 2.**
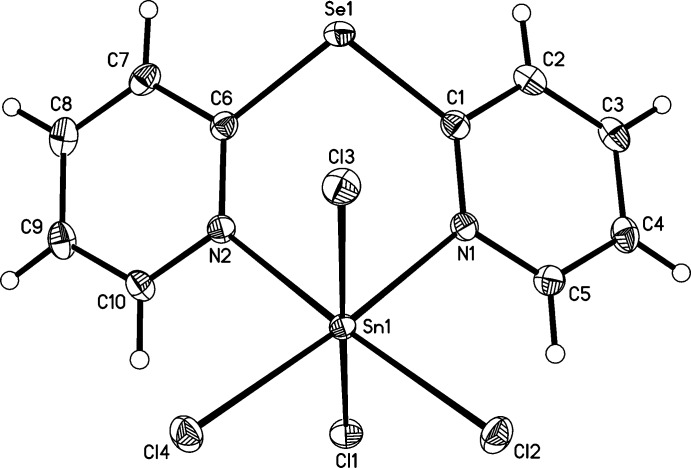
The molecular structure of I showing the atomic numbering and 50% probability displacement ellipsoids.

## supplementary crystallographic information

### Comment

2,2'-Dipyridyl sulfide plays prominent role as useful ligand in coordination chemistry (Tresoldi *et al.*, 1992; Kondo *et al.*, 1995; Blake *et al.*, 2002; Teles *et al.*, 2006; Zhao *et al.*, 2007; Wriedt *et al.*, 2008*a*, 2008*b*, 2008*c*). The important structural feature of these complexes is the practically unchangeable bond angle at sulfur atom. On the other hand, the most labile geometrical parameters in them are the dihedral angle between two pyridine rings as well as the deviation of metal atom from the mean plane of the central six-membered chelate ring passed through the two nitrogen and two carbon atoms due to the different coordination environment. It is interesting to note that 2,2'-dipyridyl selenide is also known (Dunne *et al.*, 1995), however, no structurally characterized metal complexes with this ligand were reported till now.

This article is dedicated to the first structural characterization of metal complex with 2,2'-dipyridyl selenide ligand - [bis(2-pyridyl)selenide-*k*^2^N,*N*')]tetrachlorido-tin(IV), C_10_H_8_Cl_4_N_2_SeSn (I), which was obtained by the reaction of 2,2'-dipyridyl diselenide with tin tetrachloride (Figure 1).

The molecule of I possesses overall intrinsic *C*_s_ (*m*) symmetry (Figure 2). The tin ion is coordinated by two N atoms [Sn—N 2.266 (2), 2.274 (2) Å] from bis(2-pyridyl)selenide ligand and four chloride anions [Sn—Cl 2.3717 (6)–2.3939 (6) Å] in a distorted octahedral geometry. The central six-membered chelate ring has a *boat* conformation with the Se and Sn atoms deviating from the mean plane passed through the rest four atoms of the ring at 0.692 (3) and 0.855 (3) Å, respectively. Two pyridine rings are inclined to each other with a dihedral angle of 49.62 (8)°. Remarkably, the value of the bond angle at selenium atom in I (101.51 (10)°) is almost equal to that in the free 2,2'-dipyridyl selenide ligand (101.9 (2)°) (Dunne *et al.*, 1995).

In the crystal, the molecules of I form the chains along the *a* axis by the attractive intermolecular Se1···Cl2^i^ [3.5417 (7) Å] and Se1···Cl4^i^ [3.5648 (7) Å] interactions. The chains are further linked into a three-dimensional framework by weak C—H···Cl hydrogen bonds (Table 1) and π···π stacking interactions between the pyridine rings with a centroid-centroid distance of 3.683 (3) Å. Symmetry code: (i) *x* - 1, *y*, *z*.

### Experimental

A solution of SnCl_4_ (0.13 g, 0.5 mmol) in CH_2_Cl_2_ (25 ml) was added to a solution of 2,2'-dipyridyl diselenide (0.16 g, 0.5 mmol) in CH_2_Cl_2_ (25 ml) with stirring at room temperature. After 10 min, solvent was evaporated *in vacuo.* An attempt to re-crystallization of the solid residue from CH_3_CN led to formation of the powder Se which was separated by filtration of hot solution. The filtrate was concentrated in *vacuo*. The solid was re-crystallized from CH_3_CN to give I as yellow crystals. Yield is 82%. *M*.p. = 541–543 K. ^1^H NMR (DMSO-*d*_6_, 300 MHz, 302 K): δ = 8.48 (d, 2H, J = 4.8), 7.70 (t, 2H, J = 7.8), 7.55 (d, 2H, J = 7.8), 7.28 (dd, 2H, J = 7.8, J = 4.8). Anal. Calcd for C_10_H_8_Cl_4_N_2_SeSn: C, 24.23; H, 1.63; N, 5.65. Found: C, 24.14;H, 1.59; N, 5.57.

### Refinement

The hydrogen atoms were placed in calculated positions with C—H = 0.95 Å and refined in the riding model with fixed isotropic displacement parameters [*U*_iso_(H) = 1.2*U*_eq_(C)].

### Crystal data

**Table:**

[SnCl_4_(C_10_H_8_N_2_Se)]	*F*(000) = 936
*M**_r_* = 495.63	*D*_x_ = 2.349 Mg m^−^^3^
Monoclinic, *P*2_1_/*c*	Mo *K*α radiation, λ = 0.71073 Å
Hall symbol: -P 2ybc	Cell parameters from 9615 reflections
*a* = 8.0835 (4) Å	θ = 2.2–30.0°
*b* = 12.2153 (5) Å	µ = 5.16 mm^−^^1^
*c* = 14.4710 (6) Å	*T* = 100 K
β = 101.208 (1)°	Prism, yellow
*V* = 1401.65 (11) Å^3^	0.30 × 0.24 × 0.15 mm
*Z* = 4	

### Data collection

**Table:**

Bruker SMART 1K CCD diffractometer	4096 independent reflections
Radiation source: fine-focus sealed tube	3723 reflections with *I* > 2σ(*I*)
Graphite monochromator	*R*_int_ = 0.026
φ and ω scans	θ_max_ = 30.0°, θ_min_ = 2.2°
Absorption correction: multi-scan [*SADABS*; Sheldrick, 1998)	*h* = −11→11
*T*_min_ = 0.306, *T*_max_ = 0.511	*k* = −17→17
16245 measured reflections	*l* = −20→20

### Refinement

**Table:**

Refinement on *F*^2^	Primary atom site location: structure-invariant direct methods
Least-squares matrix: full	Secondary atom site location: difference Fourier map
*R*[*F*^2^ > 2σ(*F*^2^)] = 0.025	Hydrogen site location: inferred from neighbouring sites
*wR*(*F*^2^) = 0.058	H-atom parameters constrained
*S* = 1.00	*w* = 1/[σ^2^(*F*_o_^2^) + (0.018*P*)^2^ + 5.2*P*] where *P* = (*F*_o_^2^ + 2*F*_c_^2^)/3
4096 reflections	(Δ/σ)_max_ = 0.001
163 parameters	Δρ_max_ = 1.64 e Å^−^^3^
0 restraints	Δρ_min_ = −1.10 e Å^−^^3^

### Special details

**Table:**

Geometry. All e.s.d.'s (except the e.s.d. in the dihedral angle between two l.s. planes) are estimated using the full covariance matrix. The cell e.s.d.'s are taken into account individually in the estimation of e.s.d.'s in distances, angles and torsion angles; correlations between e.s.d.'s in cell parameters are only used when they are defined by crystal symmetry. An approximate (isotropic) treatment of cell e.s.d.'s is used for estimating e.s.d.'s involving l.s. planes.
Refinement. Refinement of *F*^2^ against ALL reflections. The weighted *R*-factor *wR* and goodness of fit *S* are based on *F*^2^, conventional *R*-factors *R* are based on *F*, with *F* set to zero for negative *F*^2^. The threshold expression of *F*^2^ > σ(*F*^2^) is used only for calculating *R*-factors(gt) *etc*. and is not relevant to the choice of reflections for refinement. *R*-factors based on *F*^2^ are statistically about twice as large as those based on *F*, and *R*- factors based on ALL data will be even larger.

### Fractional atomic coordinates and isotropic or equivalent isotropic displacement parameters (Å^2^)

**Table:**

	*x*	*y*	*z*	*U*_iso_*/*U*_eq_	
Sn1	0.381830 (19)	0.755376 (12)	0.120929 (11)	0.01034 (5)	
Se1	−0.08948 (3)	0.76213 (2)	0.108418 (18)	0.01534 (6)	
Cl1	0.46143 (7)	0.84715 (5)	−0.00939 (4)	0.01648 (11)	
Cl2	0.59551 (7)	0.84559 (5)	0.23278 (4)	0.01729 (11)	
Cl3	0.27580 (8)	0.67096 (5)	0.24510 (4)	0.01741 (11)	
Cl4	0.54619 (7)	0.59645 (5)	0.10454 (4)	0.01745 (11)	
N1	0.2061 (3)	0.89921 (17)	0.12452 (14)	0.0129 (4)	
N2	0.1674 (3)	0.68416 (17)	0.01238 (14)	0.0131 (4)	
C1	0.0371 (3)	0.8955 (2)	0.11795 (17)	0.0145 (4)	
C2	−0.0569 (3)	0.9903 (2)	0.12213 (18)	0.0177 (5)	
H2	−0.1758	0.9862	0.1167	0.021*	
C3	0.0241 (3)	1.0905 (2)	0.13423 (18)	0.0182 (5)	
H3	−0.0382	1.1556	0.1387	0.022*	
C4	0.1969 (3)	1.0950 (2)	0.13974 (18)	0.0184 (5)	
H4	0.2551	1.1629	0.1477	0.022*	
C5	0.2831 (3)	0.9981 (2)	0.13332 (17)	0.0156 (4)	
H5	0.4011	1.0012	0.1352	0.019*	
C6	0.0019 (3)	0.68436 (19)	0.01536 (17)	0.0134 (4)	
C7	−0.1159 (3)	0.6267 (2)	−0.04941 (18)	0.0167 (5)	
H7	−0.2316	0.6271	−0.0450	0.020*	
C8	−0.0625 (3)	0.5688 (2)	−0.12034 (18)	0.0187 (5)	
H8	−0.1401	0.5266	−0.1639	0.022*	
C9	0.1063 (3)	0.5734 (2)	−0.12678 (17)	0.0176 (5)	
H9	0.1451	0.5369	−0.1765	0.021*	
C10	0.2175 (3)	0.6317 (2)	−0.06004 (17)	0.0153 (4)	
H10	0.3328	0.6351	−0.0651	0.018*	

### Atomic displacement parameters (Å^2^)

**Table:**

	*U*^11^	*U*^22^	*U*^33^	*U*^12^	*U*^13^	*U*^23^
Sn1	0.00923 (7)	0.01100 (8)	0.01039 (8)	0.00012 (5)	0.00091 (5)	−0.00026 (5)
Se1	0.01033 (11)	0.01823 (12)	0.01802 (12)	−0.00126 (8)	0.00412 (9)	−0.00212 (9)
Cl1	0.0171 (3)	0.0176 (3)	0.0157 (3)	−0.0012 (2)	0.0055 (2)	0.0024 (2)
Cl2	0.0152 (2)	0.0166 (3)	0.0178 (3)	−0.0017 (2)	−0.0023 (2)	−0.0027 (2)
Cl3	0.0206 (3)	0.0184 (3)	0.0138 (2)	−0.0021 (2)	0.0049 (2)	0.0021 (2)
Cl4	0.0161 (3)	0.0151 (3)	0.0203 (3)	0.0045 (2)	0.0014 (2)	−0.0015 (2)
N1	0.0133 (9)	0.0124 (9)	0.0131 (9)	0.0010 (7)	0.0025 (7)	0.0002 (7)
N2	0.0120 (9)	0.0135 (9)	0.0132 (9)	0.0004 (7)	0.0012 (7)	−0.0006 (7)
C1	0.0154 (10)	0.0161 (11)	0.0120 (10)	0.0006 (8)	0.0028 (8)	0.0000 (8)
C2	0.0177 (11)	0.0197 (12)	0.0168 (11)	0.0035 (9)	0.0058 (9)	0.0009 (9)
C3	0.0261 (13)	0.0138 (11)	0.0165 (11)	0.0055 (9)	0.0087 (10)	0.0033 (9)
C4	0.0250 (13)	0.0122 (10)	0.0184 (11)	0.0015 (9)	0.0053 (10)	−0.0007 (9)
C5	0.0160 (11)	0.0142 (10)	0.0169 (11)	0.0004 (8)	0.0038 (9)	0.0016 (9)
C6	0.0130 (10)	0.0112 (10)	0.0157 (11)	0.0003 (8)	0.0018 (8)	0.0012 (8)
C7	0.0138 (10)	0.0155 (11)	0.0187 (11)	−0.0013 (8)	−0.0018 (9)	0.0017 (9)
C8	0.0206 (12)	0.0142 (11)	0.0174 (11)	0.0022 (9)	−0.0061 (9)	0.0004 (9)
C9	0.0226 (12)	0.0159 (11)	0.0127 (11)	0.0031 (9)	−0.0007 (9)	−0.0016 (8)
C10	0.0154 (10)	0.0166 (11)	0.0138 (10)	0.0030 (8)	0.0024 (8)	−0.0021 (8)

### Geometric parameters (Å, º)

**Table:**

Sn1—N1	2.266 (2)	C2—H2	0.9500
Sn1—N2	2.274 (2)	C3—C4	1.385 (4)
Sn1—Cl3	2.3717 (6)	C3—H3	0.9500
Sn1—Cl1	2.3873 (6)	C4—C5	1.385 (3)
Sn1—Cl4	2.3901 (6)	C4—H4	0.9500
Sn1—Cl2	2.3939 (6)	C5—H5	0.9500
Se1—C6	1.910 (2)	C6—C7	1.391 (3)
Se1—C1	1.914 (2)	C7—C8	1.383 (4)
N1—C1	1.351 (3)	C7—H7	0.9500
N1—C5	1.353 (3)	C8—C9	1.387 (4)
N2—C6	1.347 (3)	C8—H8	0.9500
N2—C10	1.356 (3)	C9—C10	1.382 (3)
C1—C2	1.393 (3)	C9—H9	0.9500
C2—C3	1.383 (4)	C10—H10	0.9500
			
N1—Sn1—N2	85.14 (7)	C3—C2—H2	120.2
N1—Sn1—Cl3	89.95 (5)	C1—C2—H2	120.2
N2—Sn1—Cl3	91.02 (5)	C2—C3—C4	119.3 (2)
N1—Sn1—Cl1	85.44 (5)	C2—C3—H3	120.4
N2—Sn1—Cl1	85.43 (5)	C4—C3—H3	120.4
Cl3—Sn1—Cl1	174.40 (2)	C3—C4—C5	118.5 (2)
N1—Sn1—Cl4	174.23 (5)	C3—C4—H4	120.7
N2—Sn1—Cl4	89.11 (5)	C5—C4—H4	120.7
Cl3—Sn1—Cl4	90.68 (2)	N1—C5—C4	122.7 (2)
Cl1—Sn1—Cl4	93.59 (2)	N1—C5—H5	118.6
N1—Sn1—Cl2	90.05 (5)	C4—C5—H5	118.6
N2—Sn1—Cl2	174.98 (5)	N2—C6—C7	122.1 (2)
Cl3—Sn1—Cl2	90.42 (2)	N2—C6—Se1	123.11 (18)
Cl1—Sn1—Cl2	92.76 (2)	C7—C6—Se1	114.76 (18)
Cl4—Sn1—Cl2	95.68 (2)	C8—C7—C6	119.2 (2)
C6—Se1—C1	101.51 (10)	C8—C7—H7	120.4
C1—N1—C5	118.5 (2)	C6—C7—H7	120.4
C1—N1—Sn1	127.01 (16)	C7—C8—C9	118.8 (2)
C5—N1—Sn1	114.52 (16)	C7—C8—H8	120.6
C6—N2—C10	118.3 (2)	C9—C8—H8	120.6
C6—N2—Sn1	127.28 (16)	C10—C9—C8	119.2 (2)
C10—N2—Sn1	114.38 (16)	C10—C9—H9	120.4
N1—C1—C2	121.4 (2)	C8—C9—H9	120.4
N1—C1—Se1	123.52 (18)	N2—C10—C9	122.2 (2)
C2—C1—Se1	114.99 (18)	N2—C10—H10	118.9
C3—C2—C1	119.5 (2)	C9—C10—H10	118.9
			
N2—Sn1—N1—C1	37.1 (2)	C6—Se1—C1—C2	136.94 (19)
Cl3—Sn1—N1—C1	−53.95 (19)	N1—C1—C2—C3	−0.7 (4)
Cl1—Sn1—N1—C1	122.9 (2)	Se1—C1—C2—C3	176.52 (19)
Cl2—Sn1—N1—C1	−144.38 (19)	C1—C2—C3—C4	1.5 (4)
N2—Sn1—N1—C5	−142.22 (17)	C2—C3—C4—C5	−0.3 (4)
Cl3—Sn1—N1—C5	126.76 (17)	C1—N1—C5—C4	2.7 (4)
Cl1—Sn1—N1—C5	−56.43 (16)	Sn1—N1—C5—C4	−177.9 (2)
Cl2—Sn1—N1—C5	36.33 (17)	C3—C4—C5—N1	−1.9 (4)
N1—Sn1—N2—C6	−42.8 (2)	C10—N2—C6—C7	4.4 (3)
Cl3—Sn1—N2—C6	47.1 (2)	Sn1—N2—C6—C7	−172.03 (18)
Cl1—Sn1—N2—C6	−128.6 (2)	C10—N2—C6—Se1	−176.05 (18)
Cl4—Sn1—N2—C6	137.7 (2)	Sn1—N2—C6—Se1	7.5 (3)
N1—Sn1—N2—C10	140.65 (18)	C1—Se1—C6—N2	40.4 (2)
Cl3—Sn1—N2—C10	−129.48 (17)	C1—Se1—C6—C7	−140.06 (19)
Cl1—Sn1—N2—C10	54.85 (17)	N2—C6—C7—C8	−1.2 (4)
Cl4—Sn1—N2—C10	−38.82 (17)	Se1—C6—C7—C8	179.23 (19)
C5—N1—C1—C2	−1.4 (4)	C6—C7—C8—C9	−2.4 (4)
Sn1—N1—C1—C2	179.31 (17)	C7—C8—C9—C10	2.7 (4)
C5—N1—C1—Se1	−178.36 (18)	C6—N2—C10—C9	−4.1 (4)
Sn1—N1—C1—Se1	2.4 (3)	Sn1—N2—C10—C9	172.81 (19)
C6—Se1—C1—N1	−45.9 (2)	C8—C9—C10—N2	0.5 (4)

### Hydrogen-bond geometry (Å, º)

**Table:**

*D*—H···*A*	*D*—H	H···*A*	*D*···*A*	*D*—H···*A*
C3—H3···Cl3^i^	0.95	2.79	3.3965 (18)	122
C8—H8···Cl2^ii^	0.95	2.83	3.3126 (18)	113
C8—H8···Cl3^iii^	0.95	2.81	3.6870 (19)	154

Symmetry codes: (i) −*x*, *y*+1/2, −*z*+1/2; (ii) *x*−1, −*y*+3/2, *z*−1/2; (iii) −*x*, −*y*+1, −*z*.
